# Impact of fractionated stereotactic radiotherapy on activity of daily living and performance status in progressive/recurrent glioblastoma: a retrospective study

**DOI:** 10.1186/s13014-022-02169-1

**Published:** 2022-12-06

**Authors:** Nicolas Demogeot, J. Salleron, F. Rech, L. Taillandier, P. Royer, G. Vogin

**Affiliations:** 1 Academic Department of Radiation Therapy and Brachytherapy, Institut de Cancérologie de Lorraine– Alexis-Vautrin CLCC (Centre de Lutte Contre le Cancer – Cancer Center) – Unicancer, 6 Avenue de Bourgogne – CS 30 519, 54 511 Vandoeuvre-lès-Nancy Cedex, France; 2 Department of Biostatistics and Data Management, Institut de Cancérologie de Lorraine, Vandoeuvre-lès-Nancy, France; 3 Department of Neurosurgery, CHU de Nancy, Nancy, France; 4 Department of Neuro-Oncology, CHU de Nancy, Nancy, France; 5 Department of Radiation Oncology, Centre d’Oncologie de Gentilly, Nancy, France; 6 Centre François Baclesse, Esch-sur-Alzette, Luxembourg

**Keywords:** Glioblastoma, Recurrence, Karnofsky performance status, Cyberknife, Stereotactic radiotherapy

## Abstract

**Background:**

The prognosis of recurrent glioblastoma (GBM) is poor, with limited options of palliative localized or systemic treatments. Survival can be improved by a second localized treatment; however, it is not currently possible to identify which patients would benefit from this approach. This study aims to evaluate which factors lead to a lower Karnofsky performance status (KPS) score after fractionated stereotactic RT (fSRT).

**Methods:**

We retrospectively collected data from patients treated with fSRT for recurrent GBM at the Institut de Cancérologie de Lorraine between October 2010 and November 2017 and analyzed which factors were associated with a lower KPS score.

**Results:**

59 patients received a dose of 25 Gy in 5 sessions spread over 5–7 days (80% isodose). The median time from the end of primary radiotherapy to the initiation of fSRT was 10.7 months. The median follow-up after fSRT initiation was 8.8 months. The incidence of KPS and ADL impairment in all patients were 51.9% and 37.8% respectively with an adverse impact of PTV size on KPS (HR = 1.57 [95% CI 1.19–2.08], *p* = 0.028). Only two patients showed early grade 3 toxicity and none showed grade 4 or late toxicity. The median overall survival time, median overall survival time after fSRT, median progression-free survival and institutionalization-free survival times were 25.8, 8.8, 3.9 and 7.7 months, respectively. Initial surgery was associated with better progression-free survival (Hazard ratio (HR) = 0.48 [95% CI 0.27–0.86], *p* = 0.013).

**Conclusions:**

A larger PTV should predicts lower KPS in the treatment of recurrent GBM using fSRT.

## Background

The management of recurrent/progressive glioblastoma (GBM) is not standardized and individualized according to the general and neurological condition of the patient, relapse-free interval, recurrence pattern, anatomo-functional site of the relapse, treatments previously delivered.

The options in local pattern of recurrence/progression may include surgery, reirradiation, systemic therapy, alternating electric fields—associated with optimized supportive care within a multidisciplinary brain tumor center [1–4]. Patients who maintain a good performance status at the time of focal recurrence/progression—especially when the interval from first irradiation is longer than 6–12 months—may be candidates to localized therapy—either surgery or reirradiation according to various modalities. There is no demonstrated superiority of either approach [5, 6]—that could also be even better combined according to recent/pending studies [7, 8].

Prognostic scores have been proposed to help decision-making in recurrent/progressive GBM. The initial histology, age of the patient, time to first relapse, Karnofsky performance status (KPS), tumor volume, and extent of second surgery are integrated [9, 10]. Beyond the traditional outcome measures, it seems relevant to integrate and anticipate the quality of residual survival in patients suffering from recurrent/progressive GBM. It has been shown that different recurrent therapies did not significantly impact patients' health-related quality of life (HR-QoL) [11, 12]. However, in daily practice, it appears complicated to evaluate HR-QoL prospectively for all patients and repeatedly, especially in the advanced phase of the disease. Several methodological pitfalls have been reported [13]. To approach HR-QoL evaluation, two surrogate markers easy to collect in daily practice may be measured—i.e., the level of activities of daily living (ADL) and KPS (also prognostic of overall survival from progression) [14–17].

In a cohort of unselected consecutive patients treated homogeneously in a comprehensive cancer center, we sought to measure the impact of Fractionated Stereotactic Radiotherapy (fSRT) on ADL and KPS. We also looked for the factors that influence their deterioration.

## Methods

### Patient selection

We retrospectively selected all consecutive patients with recurrent/progressive GBM treated from October 2010 to November 2017 with fSRT as reirradiation (Cyberknife® (Accuray Inc.)) at the *Institut de Cancérologie de Lorraine*. Patient data were retrieved from the hospital database. The inclusion criteria included: in field (more than 80% of the tumour recurrence resided within the prescription 95% isodose surface), age > 18 years, de novo histologically confirmed grade IV GBM, initial treatment according to international recommendations with conventional radiotherapy (RT) fractionation, and KPS score of ≥ 60% at the time of reirradiation. The recurrence was assessed with RANO (Response Assessment in Neuro-Oncology) criteria on quarterly Magnetic Resonance Imaging (MRI) [18].

### Treatment

Only the first reirradiation was considered in this study. fSRT was delivered with 5 sessions of 5 Gy spread over 5–7 days applied on the 80% normalized isodose. The gross tumor volume was defined as the T1-enhanced lesion(s) on a brain MRI acquired within four weeks of the treatment, merged with simulation Computed Tomography (CT). An isotropic margin of 1–2 mm was added to define the Planning Target Volume (PTV). Organs at risk included brainstem, optic nerve, optic chiasm, lens, pituitary and healthy brain. Concurrent/adjuvant Chemotherapy was prescribed after reirradiation in some cases according to regional neurooncology tumor board decision.

### Follow up

After reirradiation, patients were evaluated clinically and with MRI at 1 and 3 months after the completion of reirradiation and at least every 3 months afterwards.

### Data collection

Demographic data, initial treatment characteristics and clinical data at reirradiation were retrieved from medical records. The KPS and the ADL according to the modified Barthel's index [19] were evaluated at each consultation. Headache, mobility, weight, seizure, intracranial hypertension, steroid dosage, sensorimotor defect, and topography of the tumor were recorded at the recurrence. Acute toxicity was assessed according to the Common Terminology Criteria for Adverse Events (CTCAE) v4.0 during reirradiation.

### Statistical analysis

Qualitative parameters were described as frequency and percentage. For quantitative parameters, the normality of the distribution was investigated by the Shapiro–Wilk test and was described as mean with standard deviation or median and range accordingly. Performance status impairment was defined as the loss of at least 10 points on the KPS score [17]. Patients were censored at local GBM recurrence according to RANO criteria or death, whichever came first. Prognostic factors of KPS score impairment were investigated using the univariate Cox proportional hazards model. The proportional hazards assumption were checked based on scaled Schoenfeld residuals, and the linearity assumption for continuous parameters was assessed with martingale residuals. In case of non-linearity, the parameter was dichotomized, and the thresholds were chosen according to clinical relevance. Parameters with a *p*-value less than 0.2 were tested in a multivariate Cox proportional hazards model with backward selection in order to obtain the final multivariate model. Results were explained as hazard ratio (HR) with their 95% confidence interval (95% CI). ADL decrease was defined as the time interval between the day of reirradiation to the day when ADL had decreased by 1 point [17]. Patients were censored at local recurrence according to RANO criteria or death, whichever happened first. Institutionalization-free survival corresponded to the time interval between the first fSRT session to the day of institutionalization or death, whichever happened first. Progression Free Survival (PFS) was defined as the time interval between the day of reirradiation to the day of radiological progression or death, whichever happened first. Prognostic factors of PFS were assessed using the same method as described for KPS decrease. Overall survival (OS) was measured from initial diagnosis of GBM and overall survival from the initiation of reirradiation was also measured. A sensitivity analysis was performed by excluding patients who had had a previous recurrence before the reirradiation. Prognostic factors found in the all population of this study was assessed in the subpopulation of patients that received radiotherapy for the first recurrence to check their robustness. Statistical analysis was performed using SAS software, version 9.4. (SAS Institute Inc., Cary, NC). The threshold for statistical significance was set to *p* < 0.05.

## Results

59 patients with local GBM recurrence were treated with fSRT following the initial treatment. Patient characteristics are presented in Table 1. For the primary treatment, 20 (34%), 28 (47%) and 11 (19%) patients underwent stereotactic biopsy, subtotal resection and gross total resection respectively. All patients completed the initial chemoradiation procedure. The median KPS score at recurrence was 90% (60–100%). 31 (53%) patients received systemic treatment following reirradiation (Table 1). Forty-five (76%) patients were reirradiated on the first recurrence of GBM, 10 (17%) on the second recurrence [4, 6], 3 (5%) on the third recurrence (systemic therapy) and 1 (2%) on the fourth recurrence (systemic therapy). All the patients included presented an in-field relapse—when confronting with the primary RT fields. According to the nosologic definition, only 5 patients suffered from multifocal but circumscribed relapse qualified as "non-local" relapse [20]. The reirradiation characteristics are presented in Table 2. The median time from the end of primary radiotherapy to the first recurrence was 9.5 months with 3 patients with a time inferior to 6 months (range 1.3–65 months). The median time from the recurrence diagnosis and fSRT initiation was 1 month (range 0.1–3.5 months). The median PTV of the patients included in our study was 11.4 cc. All patients completed the reirradiation course. The median follow-up appointment after fSRT initiation was at 8.8 months (range 3–54 months). Two patients were still alive at the time of analysis.

**Table 1:** Patient characteristics at diagnosis and at recurrence for the whole cohort

At diagnosis	
Age	61 ± 10
Male	32 (54)
*Surgical procedure*	
Stereotactic biopsy	20 (34)
Subtotal resection	28 (47)
Complete resection	11 (19)
*Adjuvant systemic therapy*	
Temozolomide	52 (88)
Bevacizumab	1 (2)
Nivolumab	1 (2)
Temozolomide with bevacizumab	4 (6)
Temozolomide with nivolumab	1 (2)

At recurrence	
*Number of previous recurrences*	
0	45 (76)
1	10 (17)
2	3 (5)
3	1 (2)
*KPS*	
60	1 (2)
70	10 (17)
80	9 (15)
90	35 (59)
100	4 (7)
*ADL*	
6	53 (90)
5	2 (3)
4	1 (2)
3	2 (3)
2	1 (2)
*Multifocality*	
Yes	5 (8)
No	54 (92)
*Steroids*	
Yes	14 (24)
No	45 (76)
*Antiepileptic medication*	
Yes	35 (59)
No	24 (41)
*Headaches*	
Yes	14 (25)
No	45 (75)
*Motor deficiency*	
Yes	9 (85)
No	50 (15)
*Intracranial hypertension*	
Yes	0 (0)
No	59 (100)
*Adjuvant systemic treatment after reirradiation*	31(52)
Temozolomide	12 (20)
Bevacizumab	5 (8)
Fotemustine	4 (7)
Temozolomide with bevacizumab	3 (5)
Fotemustine with bevacizumab	4 (7)
Lomustine with bevacizumab	2 (3)
Nivolumab	1 (2)

Result: mean ± standard deviation or frequency (percentage)

RTCT, concomitant adjuvant chemotherapy plus radiotherapy; KPS, Karnofsky performance status; ADL, activity of daily living

**Table 2:** Treatment characteristics of reirradiation

	Median (minimum–maximum)
Total dose (Gy)	25 (25–25)
Maximum dose at plan (Gy)	31.25 (31.13–35.71)
Number of beams	101 (56–164)
Conformity Index	1.28 (1.14–2.1)
GTV-PTV margin (mm)	2 (1–3)
PTV (cc)	11.4 (1.1–60.7)

PTV, planning target volume; GTV, gross tumor volume; cc, cubic centimeter

**Fig. 1:**
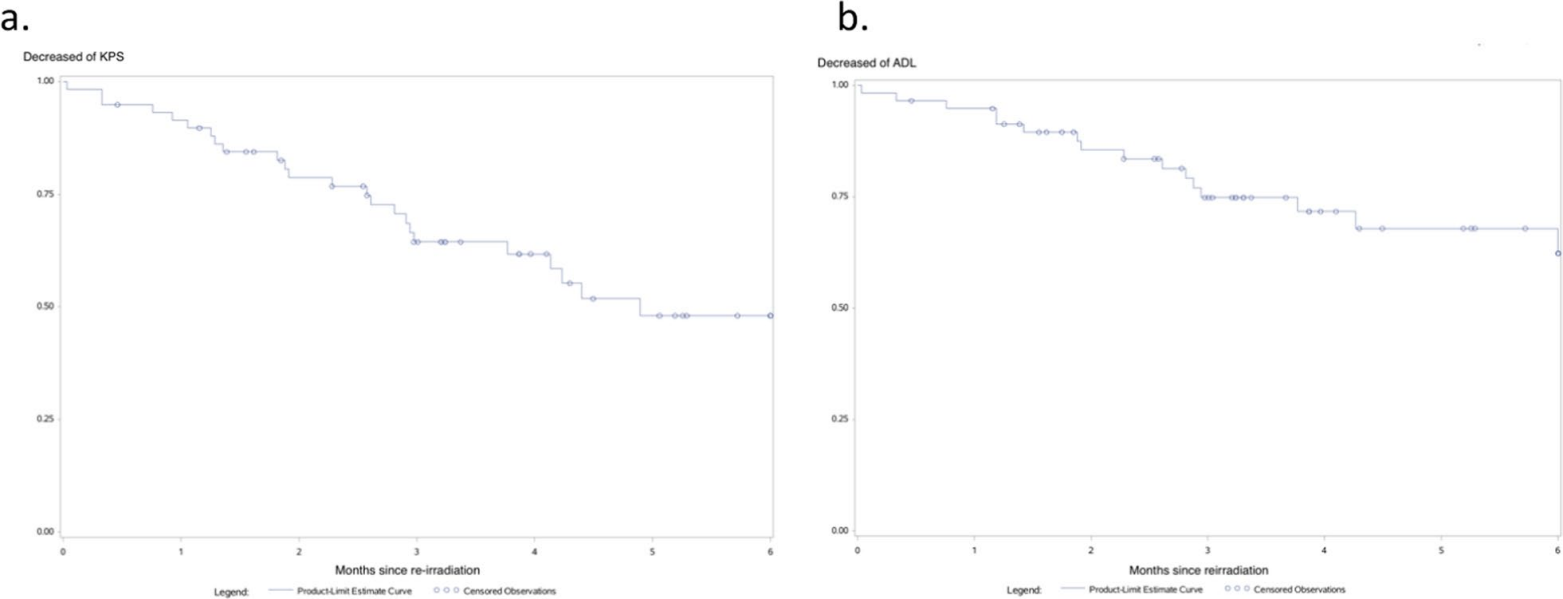
Decrease of KPS (**a**) and ADL (**b**) after fractionated stereotactic reirradiation

**Fig. 2:**
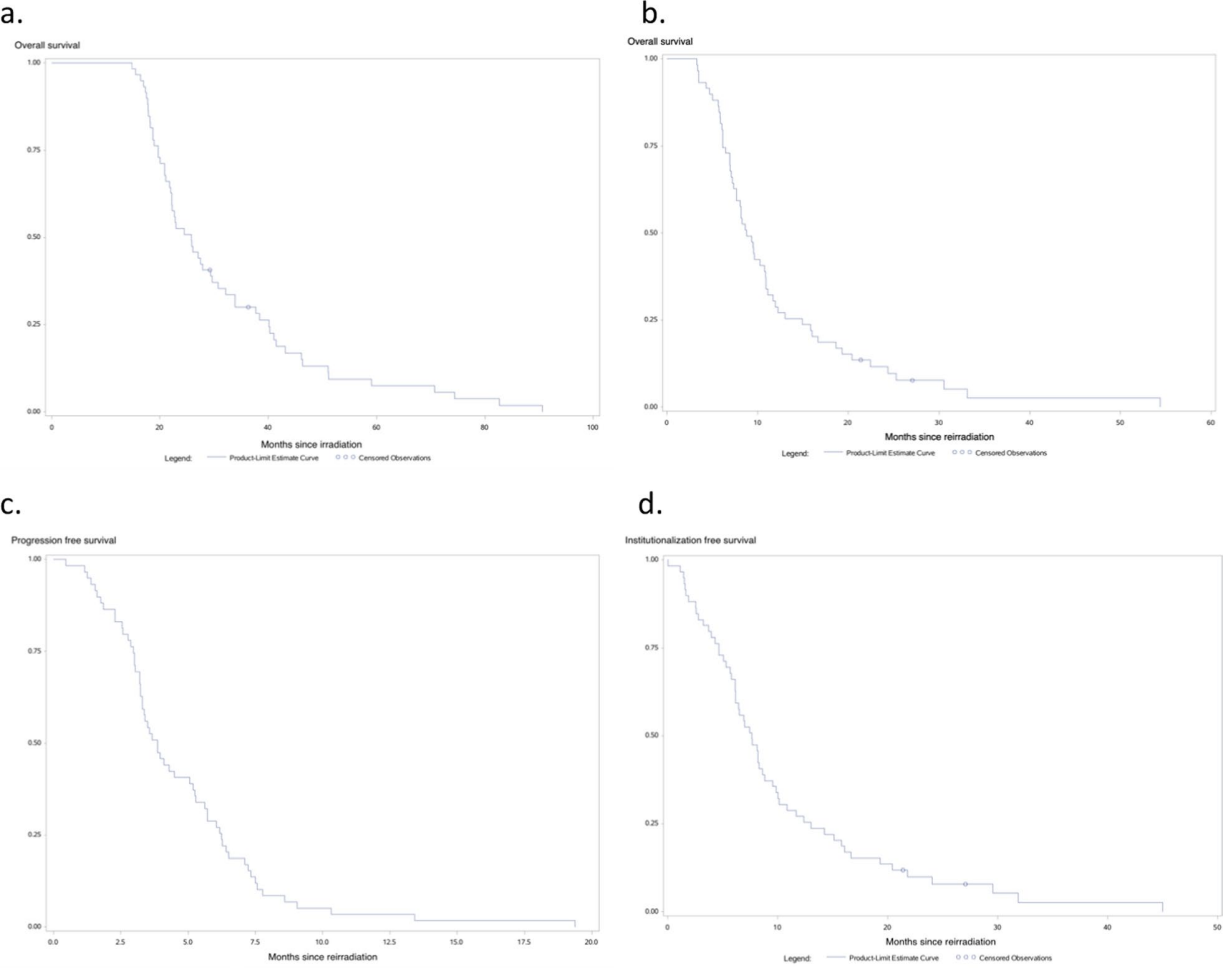
**a** Median overall survival time from diagnosis. **b** Median overall survival time from reirradiation. **c** Median progression free survival time from reirradiation. **d** Median institutionalization free survival time from reirradiation

The incidence of KPS impairment in all patients was 51.9% [95% CI 37.3–68.3] 5 months after fSRT (Fig. 1). Using univariate analysis, KPS impairment was significantly associated with headaches (HR = 2.44 [95% CI 1.07–5.59], *p* = 0.035) and a larger PTV (continuous data for an increase of 1 cc) (HR = 1.05 [95% CI 1.02–1.08], *p* = 0.016) (Table 3). A PTV value ≥ 10 cc was observed for 33 patients (55.9%) and significantly increased the risk of KPS impairment (HR 2.82 [95% CI 1.12–7.14], *p* = 0.028). "Non-local" pattern of relapse, age and time interval between first and second RT—even considered as continuous variables—did not correlate with KPS impairment (Table 3). Using multivariate analysis, only a larger PTV was associated with KPS impairment. Six months after reirradiation, 37.8% [95% CI 23.5–56.7] of patients had ADL impairment (Fig. 1). Using univariate analysis, ADL impairment was significantly associated with KPS (HR = 0.26 [95% CI 0.09–0.74], *p* = 0.011) (Table 3). The median institutionalization-free survival time was 7.7 months [95% CI 6.1–8.8] (Fig. 2). For the 45 patients who received fSRT at the first recurrence, the incidence of KPS impairment was 52.6% [95% CI 35.9–71.4] 5 months after fSRT and an increase of PTV (continuous data for an increase of 1 cc) was also significantly associated with KPS impairment (HR = 1.05 [95% CI 1.01–1.09], *p* = 0.024). Six months after reirradiation, 36.7% [95% CI 21.7–57.5] of patients had ADL impairment. Twenty-four patients experienced toxicity within three months following reirradiation: 17 patients presented with grade 1 (12 headache, 3 seizure, 1 motor defect and 1 intracranial hypertension), 5 patients experienced grade 2 (2 headache, 2 intracranial hypertension, 1 motor deficiency) and 2 patients suffered from grade 3 toxicity (2 intracranial hypertension).

**Table 3:** Prognostic factors of KPS and ADL impairment after reirradiation; univariate analysis

Variable	Hazard ratio [95% CI]	*p-*value
*KPS*		
Male gender	0.88 [0.40; 1.98]	0.763
Initial brain surgery	1.20 [0.48; 3.03]	0.697
Motor deficiency	1.32 [0.45; 3.88]	0.615
Antiepileptic medication	0.71 [0.32; 1.59]	0.405
Headaches	2.44 [1.07; 5.59]	0.035
Age (years) > 60	1.0 [0.45; 2.24]	1
Steroids (mg)	1.47 [0.64; 3.38]	0.363
PTV (cc)*	1.57 [1.19; 2.08]	0.028
KPS (%) ≥ 90	1.51 [0.60; 3.81]	0.388
Time from RTCT to fSBReRT (months) < 12	1.36 [0.59; 3.10]	0.473
Number of recurrences before reirradiation ≥ 1	1.27 [0.50; 3.20]	0.617
Multifocality	1.66 [0.49; 5.62]	0.412
*Brain topography*		
Cortical	1	
Subcortical	2.54 [1.00;6.44]	0.050
*ADL*		
Male gender	0.66 [0.24; 1.77]	0.410
Initial brain surgery	0.76 [0.26; 2.21]	0.617
Motor deficiency	2.99 [0.94; 9.54]	0.064
Antiepileptic medication	0.79 [0.29; 2.13]	0.650
Headaches	2.52 [0.91; 6.99]	0.075
Age (years) > 60	0.95 [0.35; 2.57]	0.924
Steroids (mg)	2.12 [0.78; 5.78]	0.141
PTV (cc)*	1.0 [1.0; 1.0]	0.481
KPS (%) ≥ 90	0.26 [0.09; 0.74]	0.011
Time from RTCT to fSBReRT (months) < 12	2.39 [0.77; 7.45]	0.131
Number of recurrences before reirradiation ≥ 1	1.36 [0.43; 4.30]	0.599
Multifocality	1.78 [0.36; 6.98]	0.583
*Brain topography*		
Cortical	1	
Subcortical	2.11 [0.68; 6.58]	0.196

CI, confidence interval; RTCT, concomitant adjuvant chemotherapy plus radiotherapy; fSRT, fractionated stereotactic RT

*HR computed for an increase of 10 cc

The median OS from diagnosis and from fSRT was 25.8 months [95% CI 22–29.7] and 8.8 months [95% CI 7.4–10.9], respectively (Fig. 2). The median PFS time was 3.9 months [95% CI 3.3–5.2] (Fig. 2). Using univariate analyses, surgery (HR = 0.48 [95% CI 0.27–0.86], *p* = 0.013) and steroids administration (HR = 1.81 [95% CI 1.04–3.16], *p* = 0.037) were significant associated with PFS (Table 4). Only initial stereotactic biopsy was significantly associated with PFS deterioration using multivariate analysis. For patients with treatment at the first recurrence, the median OS from diagnosis and from fSRT was 23 months [95% CI 20.9–29.7] and 10.8 months [95% CI 8.2–12], respectively. Median PFS time was 4 months [95% CI 3.4–5.7], and the initial stereotactic biopsy was significantly associated with decreased PFS (HR = 0.41 [95% CI 0.21–0.81], *p* = 0.011).

**Table 4:** Prognosis factors for decreased PFS by univariate analysis

Variable	Hazard ratio [95% CI]	*p-*value
Male gender	0.85 [0.50; 1.47]	0.566
Initial brain surgery	0.48 [0.27; 0.86]	0.013
Motor deficiency	1.42 [0.68; 2.94]	0.350
Antiepileptic medication	1.17 [0.69; 1.98]	0.567
Headaches	0.86 [0.46; 1.61]	0.645
Age (years) > 60	0.67 [0.40; 1.13]	0.135
Steroids (mg)	1.81 [1.04; 3.16]	0.037
PTV (cc) *	1.14 [0.90; 1.44]	0.269
KPS (%) ≥ 90	1.01 [0.58; 1.73]	0.985
Time from RTCT to fSBReRT (months) < 12	0.60 [0.35; 1.01]	0.055
Number of recurrences before reirradiation ≥ 1	1.84 [0.98; 3.44]	0.056
Multifocality	1.18 [0.47; 2.99]	0.715
*Brain topography*		
Cortical	1	
Subcortical	1.58 [0.77; 3.26]	0.214

CI, confidence interval; RTCT, concomitant adjuvant chemotherapy plus radiotherapy; fSRT, fractionated stereotactic RT

*HR computed for an increase of 10 cc

## Discussion

The incidence of KPS and ADL impairment in all patients were 51.9% and 37.8% respectively with an adverse impact of PTV volume on KPS. Only two patients experienced early grade 3 toxicity and none grade 4—or late toxicity. The median OS time was 25.8 months, the median OS time after fSRT was 8.8 months, and median PFS and institutionalization-free survival times were 3.9 and 7.7 months, respectively.

KPS score is a major prognostic marker in neurooncology [21]. A larger PTV was adversely correlated, which is probably due to the reirradiation of a larger amount of healthy brain. To our knowledge, this study is the first to document the changes in KPS score after fSRT and the related prognostic factors. In our study, the median institutionalization-free survival time was 7.7 months [95% CI 6.1–8.8]. It was consistent with the ADL impairment which was correlates with KPS at recurrence. In this study, KPS impairment did not lead to an ADL impairment and an institutionalization with dependency on caregivers. This can be explained by the minor KPS impairment (only 10 points for the majority of patients) after radiotherapy. Only two patients experienced early grade 3 toxicity and none grade 4 or late toxicity—confirming the acceptable tolerance profile of fSRT in the treatment of GBM recurrence/progression [8, 22–29].

The median PFS was comparable to those previously reported [5, 22–36]. The timing of fSRT reirradiation according to disease progression did not really impact PFS or OS. Two systematic reviews and meta-analysis meeting our criteria were recently published including one that specifically assessed CyberKnife® excluding anaplastic tumors like ours [5, 36]. Most studies referred are single-center case series. Our study has the second largest population reported. As for us, the Gross Tumor Volume (GTV) at recurrence was defined as the MRI gadolinium-enhanced area and the PTV was reconstructed adding 0 to 3 mm margin to the GTV. Amino-acid PET has also been proposed to tailor GTV delineation [37]. The median PTV volume was 12.1 cc. The median number of fractions was 3 (range 1–6) and the median dose was 24.5 Gy (range 13.9–48.8). The prescribed marginal isodose ranged from 78 to 91%. In their meta-analysis, Kazmi et al. recommend a highly conformal technique with a hypofractionated regimen (such as 25 Gy in 5 fractions or 35 Gy in 10 fractions), taking into account the volume and location of the recurrent tumor. The authors were unable to meta-analyze the effect of Cyberknife treatment on KPS, cognitive function, and quality of life.

Our study had comparable OS with other anterior studies addressing local management of GBM local recurrence [5, 22–36, 38]. Non-surprisingly, the median OS was better than the seminal publication of Stupp et al. which testifies that only a sub proportion of relapsing GBM patients (younger patients with good KPS and local relapse) are eligible for a second local treatment [2, 39, 40].

Our study had several strengths. First, we had a large homogeneous population with one histology and one treatment schedule for all patients. Second, this study is the first to document the changes in KPS and ADL score after fSRT and the related prognostic factors of KPS impairment. Third, the standardized medical records have allowed a rigorous collection of medical data. The median PTV of the patients included in our study is relatively low compared with other studies [5, 22–36]—which can reduce the bias of the volume effect on symptomatology whatever the etiology.

As already mentioned, in this retrospective real-life study, we included consecutive patients we considered eligible for reirradiation according to international recommendations. This therefore presupposed sorting out the cases of local relapse and it was not possible to obtain a perfectly homogeneous cohort on the quality criteria of the initial surgery or nature of the second-line and subsequent chemotherapy. We concentrated efforts on the decision-making criteria and related thresholds most reported in the literature (KPS at relapse, pattern of relapse, time interval between irradiations) [2, 40–42]. We report minor deviations on these major criteria. However, our inclusion criteria remain broadly comparable to those of previous studies. We did not accrue patients with meningeal involvement or direct contact with the ventricle system at recurrence [20, 43]. Due to the retrospective design of our study, we could not systematically retrieve all the prognostic factors recently identified—e.g. Isocitrate dehydrogenase (IDH)-1/2 mutation, and O6-methylguanine DNA methyltransferase (MGMT) promoter methylation [8, 44–47]. Although we have used the RANO classification to assess response, we must acknowledge that it may be difficult to differentiate post-treatment effects from genuine recurrence/progression—which could have impacted the selection of patients and results [40, 48]. Finally regarding the dose-fractionation schedule we chose, optimum dose and technique of reirradiation has yet to be established [2, 40, 49].

In a recent study, high-dose salvage re-irradiation in carefully selected patients with recurrent/progressive glioma was associated with stable HR-QoL (preserved functional domains and reduced symptom burden) and improvement in ADL (greater functional independence) over time with encouraging survival outcomes [12]. This finding allowed us to propose a prudent reirradiation dose escalation in the hope of increasing the medical benefit—especially when the PTV is circumscribed. Maitre et al. confirmed that easy-to-collect indicators may help the clinician to better appreciate the therapeutic options in recurrent/progressive GBM. To go further in this investigation, it might be tempting to approach assessment of instrumental daily living (IADL) despite cognitive deficits observed in these patients [50]. The E*uropean Organisation for Research and Treatment of Cancer* (EORTC) Quality of Life Group is currently validating a specific questionnaire [51].

## Conclusion

As previously meta analyzed, we confirm that fSRT shows a favorable therapeutic ratio in the treatment of recurrent GBM—especially in selected situations when (cumulative) PTV volume is restricted. Due to a linear correlation between PTV volume and we tried to define a threshold but the benefit is linked to the PTV with a continuous value. In this complex palliative situation, easy-to-collect surrogate markers of HR-QoL data could be used to support decision-making when multiple treatment options are available, in addition to the published re-irradiation risk scores. Future prospective studies included patient-reported outcomes are needed, since KPS and ADL are only a reflection of HR-QoL.

## Data Availability

The datasets used and/or analysed during the current study are available from the corresponding author on reasonable request.

## Abbreviations

ADL: Activities of daily living
CT: Computed tomography
CTCAE: Common terminology criteria for adverse events
EORTC: European Organisation for Research and Treatment of Cancer
fSRT: Fractionated stereotactic radiotherapy
GBM: Glioblastoma
GTV: Gross tumor volume
HR: Hazard ratio
HR-QoL: Health-related quality of life
IADL: Instrumental activities of daily living
IDH: Isocitrate dehydrogenase
KPS: Karnofsky performance status
MGMT: O6-methylguanine DNA methyltransferase
MR: Magnetic resonance imaging
OS: Overall survival
PFS: Progression free survival
PTV: Planning target volume
RANO: Response assessment in neuro-oncology
RT: Radiotherapy
